# Efficacy of CRISPR-Based Gene Editing in a Sickle Cell Disease Patient as Measured through the Eye

**DOI:** 10.1155/2022/6079631

**Published:** 2022-08-22

**Authors:** Alexander Pinhas, Davis B. Zhou, Oscar Otero-Marquez, Maria V. Castanos Toral, Justin V. Migacz, Jeffrey Glassberg, Richard B. Rosen, Toco Y. P. Chui

**Affiliations:** ^1^Department of Ophthalmology, New York Eye and Ear Infirmary of Mount Sinai, New York, NY, USA; ^2^Department of Ophthalmology, Icahn School of Medicine at Mount Sinai, New York, NY, USA; ^3^Departments of Emergency Medicine,Hematology and Medical Oncology, Icahn School of Medicine at Mount Sinai, New York, NY, USA

## Abstract

Sickle cell disease (SCD) exists on a phenotypic spectrum with variable genetic expressivity, making it difficult to assess an individual patient's risk of complications at any particular point in time. Current and emerging SCD treatments, including CRISPR-based gene editing, result in a variable proportion of affected red blood cells (RBCs) still vulnerable to sickling. Clinical serological indicators of disease such as hemoglobin, indirect bilirubin, and reticulocyte count and clinical metrics including number of emergency department visits and hospitalizations over time often fall short in their ability to objectively quantify ischemic disease activity and efficacy of treatments. Clearly, better clinical biomarkers are needed. The rapidly developing field of oculomics leverages the transparent nature of the ocular tissue to directly study the retinal microvasculature in order to characterize the status of systemic diseases. In this case report, we demonstrate the ability of optical coherence tomography angiography (OCT-A) to detect and measure micro-occlusive events within the retinal capillary bed before and after RBC exchange transfusion and following CRISPR-based gene editing, as an indicator of systemic ischemic disease activity and measure of treatment efficacy. The implications of these findings are discussed.

## 1. Introduction

Sickle cell disease (SCD) is the most common inherited blood disorder in the world and causes significant body pains, morbidity secondary to ischemic end-organ damage, and mortality at a relatively young age [1]. Evidence suggests that SCD exists on a phenotypic spectrum with variable genetic expressivity, making it difficult to stratify risk of complications for a given individual [2]. Even with current and emerging SCD treatments, including CRISPR-based gene editing of bone-marrow-derived hematopoietic stem cells, studies have shown that a variable degree of phenotypic mosaicism persists, with a variable proportion of red blood cells (RBCs) still able to sickle and cause disease [3].

Current indicators of disease severity and treatment response used in clinic and in clinical drug trials include serological biomarkers of hemolytic anemia and number of vaso-occlusive crises (VOCs), emergency room visits, and hospitalizations over time [4]. These clinical indicators lack the ability to immediately and objectively measure ischemic disease activity in SCD patients before or after treatment, which limits their ability to stratify patients according to prognosis and risk [5]. Improved biomarkers are clinically needed to help assess disease activity and response to therapy [6].

Oculomics, a rapidly developing field dedicated to identifying ocular biomarkers for systemic disease, is gaining attention for several cardiovascular and neurological applications [7]. A variety of retinal microvasculature features have become recognizable for their associations with systemic disease states [8]. Optical coherence tomography angiography (OCT-A) has emerged as a powerful clinical tool to image the retinal capillary bed, as it provides higher transverse and axial resolution than conventional color fundus photography or intravenous fluorescein angiography [9,10].

OCT-A allows direct visualization of dynamic occlusive events of the microvasculature secondary to SCD within the macula. Quantitative biomarkers using sequential OCT-A scans, measuring macular capillary density and intermittent perfusion indices, provide an indication of systemic SCD activity and treatment response [11]. Recently, adaptive optics scanning light ophthalmoscopy (AO-SLO) has been used for *in vivo* study of intermittent perfusion events detected by OCT-A and has revealed the role of sickled RBCs in promoting intravascular thrombus formations [12].

The purpose of this case report was to demonstrate the use of OCT-A in an SCD patient in assessing disease activity and the impact of treatment with RBC exchange transfusion therapy versus CRISPR-based gene editing. BCL11A has been identified as a potent repressor of *γ*-globin expression and hemoglobin F (HbF) production in adult erythrocytes [13]. The patient in this case report underwent CRISPR-based gene editing targeting the *BCL11A* gene with the purpose of disease modification through HbF elevation.

### 1.1. Case Description

A 23-year-old male had severe HbSS disease diagnosed at birth with an extensive history of complications. His sickle cell anemia required chronic monthly RBC transfusions and multiple RBC exchange transfusions (approximately 100 transfusions over his lifetime), resulting in hemochromatosis necessitating oral iron chelation therapy. Other SCD complications included frequent VOCs with hypoxia and fevers, multiple episodes of acute chest syndrome (ACS; intubated once at age 9), two episodes of pneumonia, one episode of hyperhemolytic crisis, recurrent cellulitis from oxacillin-susceptible *Staphylococcus aureus*, nocturnal enuresis, avascular necrosis of both hips and left shoulder, history of priapism, and a history of cholecystitis status post cholecystectomy at age 15. His history was negative for clinical stroke. The patient averaged 6–8 emergency room visits per year over the 2 years leading up to gene editing, for which he was usually admitted. Occasionally, he would receive intravenous pain medications in the clinic as well.

The patient's past ocular history was significant for high myopia (OD -6.0D and OS -6.75D), dry eye disease, and nonproliferative sickle cell retinopathy of both eyes. His family history was significant for both parents with sickle cell trait and a brother with HbSS disease.

The patient's medications included L-glutamine 15 g two times a day, folic acid 1 mg daily, and deferasirox 1.8 g daily. The patient required an occasional albuterol but did not meet the clinical criteria for asthma. His pain control medications included gabapentin 100 mg three times a day, ibuprofen 600 mg every 6 hours as needed for pain, dilaudid 4 mg every 4 hours as needed for pain, and oxycodone-acetaminophen 10–325 mg every 4 hours as needed for pain. The patient required 15–30 tablets of dilaudid per month and 20–30 tablets of oxycodone-acetaminophen per month for pain control.

The patient had a history of treatment with hydroxyurea 2 g/2.5 g, alternating every other day, starting from age 13. The patient tolerated hydroxyurea well and maintained a good serological response to it, as evidenced by his average % HbF of 20% while on hydroxyurea. However, clinically, the patient's severe cycles of VOCs and hemolytic anemia continued, and the decision was made to pursue further investigational treatment with gene editing. Thus, in preparation for gene editing, the patient's hydroxyurea was discontinued and the patient underwent gene editing approximately 1 year later.

During a hematology clinic visit a few days prior to his first OCT-A imaging visit, the patient described continued limitation in his physical abilities, complaining of shortness of breath and hip pain with moderate physical exertion, such as climbing a few flights of stairs. He denied brain fog or limitations in his mental capacity. His vital signs included a blood pressure of 109/65 mmHg, a heart rate of 91 beats per minute, a temperature of 37.3°C (99.1°F) (oral), respirations of 17 breaths per minute, a blood oxygen saturation of 94% on room air, a weight of 79.1 kg (174 lb 6.4 oz), a height of 5′9″, and a body mass index of 25.02 kg/m^2^. On physical exam, he had tenderness over his hip joints and limited range of motion with pain in both hips. Relevant serological values within 1 year before gene editing (range of values from when patient was not taking hydroxyurea and was awaiting gene editing) and at 5 months after gene editing (single time point) are shown in Table 1.

His eye exam on the day of his first OCT-A imaging showed a best-corrected visual acuity with corrective spectacles of 20/20 in both eyes and intraocular pressures of 15 and 14 mm Hg in the right and left eye, respectively. His anterior segment exam was significant for decreased tear breakup time and scleral pallor, and was otherwise unremarkable. Dilated fundus examination and ultrawide-field fluorescein angiography revealed temporal peripheral nonperfusion without evidence of neovascularization (Optos California icg, Optos PLC, Dunfermline, United Kingdom). OCT macula showed mild temporal macular thinning (Heidelberg Spectralis HRA + OCT, Heidelberg Engineering Inc, Heidelberg, Germany).

Three months prior to and in preparation for gene editing, the patient's frequent RBC transfusions were replaced with RBC exchange transfusions. The patient was imaged longitudinally with OCT-A during 3 serial visits using the protocol described below. His first imaging visit took place 2 months after receiving his initial exchange transfusion. His second imaging visit occurred 1 month later, which was 2 weeks after his second exchange transfusion. The patient underwent a CRISPR-based gene editing procedure 1 month after his second imaging visit. His third imaging visit was 7 months after his second and approximately 6 months after his gene editing.

At a hematology clinic visit 5 months following gene editing, the patient reported that he was no longer experiencing shortness of breath with physical exertion; however, he reported persistent hip pains. His vital signs at that visit were a blood pressure of 116/77 mm Hg, a heart rate of 94 beats per minute, a temperature of 36.1°C (97°F) (tympanic), respirations of 19 breaths per minute, a blood oxygen saturation of 96% on room air, a weight of 77.1 kg (170 lb), a height of 5′9″, and a body mass index of 25.1 kg/m^2^.

The patient had no SCD complications in the 6 months following gene editing. Due to persistent hip pains from avascular necrosis, the patient was scheduled for hip replacement. He was less reliant on his pain control medications following gene editing but they were continued mainly for his hip pain and included gabapentin 100 mg three times a day, ibuprofen 600 mg every 6 hours as needed for pain, and oxycodone-acetaminophen 10–325 mg every 4 hours as needed for pain. The patient was continued on L-glutamine 15 g two times a day, folic acid 1 mg daily, and deferasirox 1.8 g daily.

## 2. Methods

Prior to gene editing, the patient was recruited to participate in the imaging research study using OCT-A to quantify systemic SCD severity and treatment efficacy. The study was conducted at the New York Eye and Ear Infirmary of Mount Sinai. It followed the tenets of the Declaration of Helsinki and was HIPAA compliant and was approved by the Institutional Review Board of New York Eye and Ear Infirmary of Mount Sinai. Written informed consent was obtained from the patient after an explanation of the nature and risks of the study. The left eye was randomly chosen as the eye of interest in this patient.

### 2.1. OCT-A Image Acquisition

Mydriasis and cycloplegia of the left eye were induced at the beginning of each imaging visit, with 1 drop each of 2.5% phenylephrine hydrochloride ophthalmic solution (Bausch & Lomb Inc., Tampa, FL) and 1% tropicamide ophthalmic solution (Akorn Inc., Lake Forest, IL).

The OCT-A imaging protocol was performed at each of the three imaging visits as previously described [11, 14], using a commercial SD-OCT system with the illumination source centered at 840 nm, a bandwidth of 45 nm, an axial resolution of 5 *µ*m, and an acquisition rate of 70,000 A-scans per second (Avanti RTVue-XR; AngioAnalytics software version 2017.1.0; Optovue, Fremont, California, USA). Each imaging visit consisted of 2 sessions, 1 hour apart. During both sessions of each imaging visit, 10 sequential 3 × 3 mm en face OCT-A scans centered on the fovea were acquired.

### 2.2. OCT-A Image Processing

The OCT-A scans were segmented to include the full vascular slab, extending from the inner limiting membrane to 9 *µ*m below the posterior boundary of the outer plexiform layer, in order to include the superficial, intermediate, and deep capillary plexuses. Each set of 10 sequential OCT-A scans was registered and averaged using ImageJ (ImageJ, US National Institutes of Health, Bethesda MD, USA) [15]. Thus, each of the three imaging visits resulted in two averaged images, one from the first session and one from the second session 1 hour later. The foveal avascular zone (FAZ) and noncapillary vessel areas were then removed by thresholding from each averaged OCT-A (Adobe Photoshop CS6, Adobe Systems, Inc., San Jose, CA, USA; MATLAB 2018b; MathWorks, Natick, MA).

For each visit, between-session pixel intensity differences were calculated by subtracting the first session averaged OCT-A from that of the second session. Pixels at which the difference in pixel intensity was outside the normal range (0.01^st^–99.99^th^ percentile) of between-session pixel intensity variations were defined as regions with significant intermittent perfusion. Capillaries that were perfused in the averaged OCT-A from the first imaging session that then lost perfusion at the 1-hour follow-up imaging session were labeled nonperfused. Conversely, capillaries not visible during the first imaging session which appeared in the 1-hour follow-up session were labeled reperfused. The between-session intermittent perfusion index (IPI) was defined as the percent of the entire OCT-A scan (minus FAZ and noncapillary vessel area) demonstrating either nonperfusion or reperfusion.

Intermittent perfusion maps were created to highlight nonperfused and reperfused capillary segments. Segments demonstrating subsequent nonperfusion were highlighted in red, and those exhibiting subsequent reperfusion were highlighted in cyan.

## 3. Results and Discussion

**Figure 1:**
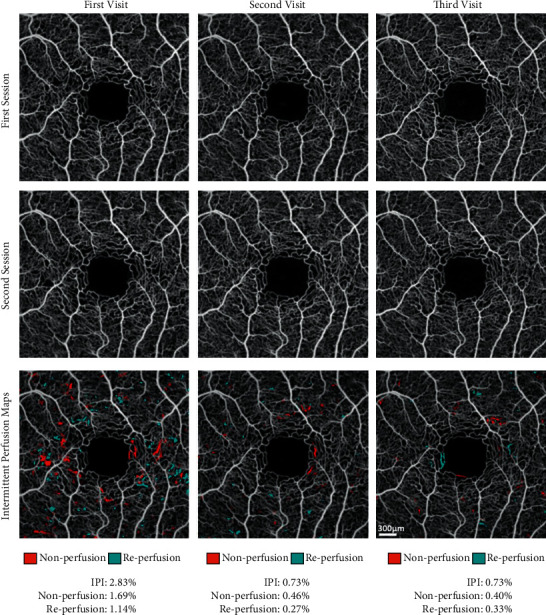
OCT-A images of the left eye of the HbSS patient, imaged across 3 imaging visits. The left column shows averaged OCT-A images from the first visit, the middle column from the second visit, and the right column from the third visit. The first and second rows show the averaged OCT-A images from the first and second sessions, respectively, taken 1 hour apart during each visit. The third row of images represents the computed intermittent perfusion maps. Nonperfusion between sessions is highlighted in red, and reperfusion between sessions is highlighted in cyan. The sum of the nonperfusion and reperfusion percent densities equals the intermittent perfusion index (IPI).

At the first OCT-A imaging visit, the patient was 2 months after his first of the two exchange transfusions in preparation for gene editing. OCT-A showed a relatively high disease activity, with an IPI of 2.83%, a nonperfusion area of 1.69%, and a reperfusion area of 1.14% (Figure 1, Column 1). These results suggested that the effects of the initial exchange transfusion were wearing off. One month later, 2 weeks after the second exchange transfusion, the second imaging session showed that IPI had improved substantially to 0.73%, with a nonperfusion area of 0.46% and a reperfusion area of 0.27% (Figure 1, Column 2). This substantial improvement in IPI reflected the expectation that most of the circulating blood 2 weeks after exchange transfusion was healthy donor blood.

The patient underwent CRISPR-based gene editing 1 month after his second imaging visit. The patient did not undergo any repeat blood transfusions after undergoing gene editing. OCT-A images from the third imaging visit, approximately 6 months after gene editing and 7 months after the second imaging visit, showed an IPI of 0.73%, with a nonperfusion area of 0.40% and a reperfusion area of 0.33% (Figure 1, Panel 3). Thus, CRISPR-based gene editing achieved a reduction in microvascular occlusive events that was comparable to exchange transfusion, but without the need to continually repeat therapy. Fundus photography and OCT repeated at all 3 visits did not show appreciable change.

Our case report demonstrates how OCT-A can be used to measure systemic SCD disease activity and response to treatment noninvasively through the visualization and measurement of dynamic vaso-occlusive episodes in the retinal capillary bed. This is the first report documenting macular OCT-A before and after gene editing in SCD. Our results are consistent with the prior paper by Zhou et al. establishing this imaging methodology [11]. In that paper, the IPI of all SCD patients (*n* = 13; including 1 sickle cell trait patient without treatment, 1 HbSC patient without treatment, 3 HbSS patients without treatment, 4 HbSS patients on hydroxyurea, and 1 HbSS patient after gene editing) was 0.36 ± 0.72% (median ± interquartile range). Subgroup analysis showed that IPI was 2.59 ± 1.00% for the HbSS patients without treatment and 0.52 ± 0.39% for the HbSS patients on hydroxyurea. Zhou et al. also reported an IPI of 3.0% in a treatment-naïve HbSS patient, which was reduced to 0.5% following two months of oral hydroxyurea therapy. For comparison, the IPI of unaffected controls was found to be 0.01 ± 0.03%. Since oral hydroxyurea therapy and gene editing targeting the *BCL11A* gene are both disease-modifying treatments based on HbF induction, it is interesting to compare the similar IPI values between the HbSS patients on hydroxyurea from the previous publication and our patient after gene editing (0.52% ± 0.39% versus 0.73%, respectively). Of note, the HbSS patient after gene editing from the previous publication had a reported IPI of 0.16%, although the baseline IPI prior to gene editing was unknown. It is thus difficult to draw conclusions on the relative effectiveness of treatments from these results given the multiple limitations, including limited sample sizes and lack of detail on patients' serologies and disease courses in the prior publication. Future studies combining traditional biomarkers with OCT-A need to elucidate the relative effects of various treatments and combinations of treatments in SCD, including hydroxyurea, blood transfusions, and gene editing.

SCD is a complex and multifactorial disease process that begins with polymerization of HbS and deformation of sickled RBCs, initiating rheological, inflammatory, and cellular events leading to cycles of vaso-occlusion and hemolytic anemia [5]. Conventional biomarkers of disease fall short in objectively measuring immediate ischemic disease activity in SCD. Serological biomarkers of hemolytic anemia, such as hemoglobin, hematocrit, indirect bilirubin, and reticulocyte count, correlate to episodes of VOCs; however, they record their hemolytic aftermath and not the VOCs themselves. The number of VOCs and hospitalizations over time attempt to estimate ischemic disease burden over months to years but lack the granularity to measure the severity and dynamics of vaso-occlusions occurring at any particular point in time.

In our patient, prior to gene editing, serologies indicated significant hemolytic anemia (Hgb 7.7–9.6 g/dL, indirect bilirubin 2.7–8.1 mg/dL, and reticulocyte count 7.0–10.2%). There was evidence of significant ischemic disease burden, as he averaged 6–8 emergency room visits per year for VOCs and ACSs and was dependent on opiate medications for pain control. Five months following gene editing, hemolytic anemia was significantly improved (Hgb 13.3 g/dL, indirect bilirubin 3.3 mg/dL, and reticulocyte count 4.7%). It was difficult to measure the degree of ongoing subclinical ischemic burden since the patient was no longer exhibiting clinical phenomena of VOCs or ACS and was using opiate medications for pain control for his avascular necrosis of the hip.

The severity of SCD micro-occlusive activity, as measured by OCT-A at the first and third imaging visits, was in concordance with the severity of ongoing hemolytic anemia evident on serologies before and after gene editing. OCT-A provided a more sensitive tool for measuring the subclinical vaso-occlusive events than measures dependent on clinically evident VOC and ACS.

This case confirms that CRISPR-based gene editing in SCD, while substantially effective, may not completely eliminate vaso-occlusive activity. This is consistent with prior reports that show that the success of gene editing varies between individuals and gene editing results in a phenotypic spectrum [3]. What was unclear in our case, since % HbS after gene editing was 50%, is whether the continued vaso-occlusive events in our patient were due to residual circulating sickled RBCs, persistent dysfunction of the endothelium and coagulation cascade, or a combination of both. Future studies using AO-SLO along with OCT-A may help answer this question. The clinical significance of residual vaso-occlusive episodes measured on OCT-A following gene editing in SCD also remains unclear and will need to be studied prospectively.

Noninvasive ocular biomarkers such as IPI have the potential to allow hematologists to be less reliant on blood draws and measures such as number of VOCs and hospitalizations over time to grade ischemic disease activity, measure disease progression, prognosticate risk of systemic and ophthalmic complications, and measure treatment response objectively and quantitatively. OCT-A may be useful for minimizing time to decision making to maintain or alter therapy, including identifying candidates for adjunctive oral therapy following gene editing, which can decrease patient morbidity and mortality while reducing healthcare expenditures.

## Figures and Tables

**Table 1:** Serologies before and after gene editing.

Serology	Before gene editing	After gene editing	Normal range
Hb	7.7–9.6 g/dL	13.3 g/dL	13.9–16.3 g/dL
% Hb A	45%	0.00%	N/A
% Hb S	70–80%	50%	N/A
% Hb F	1.9%	45%	N/A
Hct	23–27.5%	36%	42.0–52.0%
MCV	88.7–109.2 FL	95.7 FL	80.0–98.0 FL
RDW	22.9–24.1%	15%	11.5–15.0%
Indirect bilirubin	2.7–8.1 mg/dL	3.3 mg/dL	0.2–0.8 mg/dL
Reticulocytes	7.0–10.2%	4.70%	0.7–2.8%

Hb, hemoglobin; %, percent; Hct, hematocrit; MCV, mean corpuscular volume; RDW, red blood cell distribution width; *g*, grams; dL, deciliter; N/A, nonapplicable; FL, femtoliter; mg, milligram.

## Data Availability

The clinical data and the OCT-A data used to support the findings of this study are included within the article.
